# Adaptations in Visual Search Behaviour as a Function of Expertise in Rugby Union Players Completing Attacking Scenarios

**DOI:** 10.3389/fpsyg.2022.837558

**Published:** 2022-03-31

**Authors:** Kjell N. van Paridon, J. Lally, P. J. Robertson, Itay Basevitch, Matthew A. Timmis

**Affiliations:** ^1^Cambridge Centre for Sport and Exercise Science, Anglia Ruskin University, Cambridge, United Kingdom; ^2^School of Social and Behavioral Sciences, Northcentral University, San Diego, CA, United States

**Keywords:** visual search behaviour, eye tracking, rugby, representative design, expertise

## Abstract

The current study investigated the adaptations which occur in visual search behaviour as a function of expertise in rugby union players when completing attacking scenarios. Ten experienced players (EP) and ten novice players (NP) completed 2 vs. 1 attacking game scenarios. Starting with the ball in hand and wearing a mobile eye tracker throughout, participants were required to score a try against a defender. The scenarios allowed for a pass to their supporting player (Spin Pass or Switch) or trying to run past the defender (Take-Player-On or Dummy Switch). No between group differences were found in fixating on the supporting attacking player (*p* > 0.05). However, EP increased the length (*p* = 0.008) and frequency (*p* = 0.004) looking at the area immediately ahead of the supporting player, particularly when executing a spin pass. NP fixated longer (*p* = 0.005) and more frequently (*p* = 0.032) at the defender, whilst EP fixated more frequently in the space the supporting player would run into in Switch and Dummy Switch scenarios (*p* = 0.025). More successful passes were completed and tries scored by EP compared to NP (*p* = 0.001). Differences in visual search behaviour between experienced and NP suggest that the experts extract information from areas directly related to guiding the motor action; the space immediately ahead of the support player to pass the ball in. Contrastingly, novices use a more allocentric perspective where the actions from the defender are used to guide their motor actions.

## Introduction

Due to the experiences players gather through deliberate practice and competitive match play, they develop a task-specific knowledge base which creates an opportunity to interpret events encountered in sporting situations with reference to those previously experienced (Williams, 2000). This knowledge base results in domain-specific adaptations in long term working memory, facilitating sophisticated strategic or tactical problem representations (McPherson, 1999, 2000; McPherson and Kernodle, 2007). In team sports, skilled players have superior strategic awareness compared to novices (i.e., soccer, Williams, 2000), which impacts player positional location (González Víllora et al., 2013) and the ability to predict future movements of others in the game (Brault et al., 2010, 2012).

A player’s strategic awareness is linked to the visual information they acquire from within the environment. For example, through watching filmed based scenarios in soccer, when anticipating pass direction, experienced players (EP) generally look more peripherally in the display, fixating on other players or areas of space that could be exploited and demonstrate a higher search rate (more fixations of shorter duration) compared to novice players (NP) who fixate the player with the ball (e.g., Helsen and Pauwels, 1992, 1993; Williams et al., 1994, 1999; Helsen and Starkes, 1999; Vaeyens et al., 2007). These adaptations in visual search behaviour result in the EP being more accurate in anticipating scenario outcome compared to NP in video-based paradigms.

Physically executing a task, results in a different visual search behaviour compared to when little/no motor component is required (e.g., watching a video Land and Hayhoe, 2001; Dicks et al., 2010). This adaptation of visual search behaviour can be attributed to the presence of two-visual systems; a dorsal and ventral stream (Goodale and Milner, 1992; van der Kamp et al., 2008). The ventral stream creates an allocentric representation of the world where the initial constraints of the action are defined. In contrast, the dorsal stream provides an egocentric perspective mainly associated with identifying contrast and movement related to the execution of the motor action. The separation of these pathways is debated (see Mann et al., 2021) but instigated the use of more representative experimental designs. When investigating visual search behaviour in sport, and to avoid decoupling of perception of information from the motor action (c.f. Mann et al., 2010) it is important to use representative designs (c.f. Araujo et al., 2007). If participants engage with physically executing the task(s) it helps to ensure that information is derived from both ventral and dorsal streams. This is of particular importance in self-paced tasks with varying environmental constraints where differences in expertise are examined. Expertise differences are evident in superior motor actions suggesting an enhanced ability to rely on information from the dorsal stream. This is reflected in findings that differences in performance and visual search behaviour between experts and novices disappear when simulated or uncoupled actions are used in comparison to in-situ tests (van der Kamp et al., 2008; Mann et al., 2021). In other words, expertise differences might be less apparent when ventral stream information is derived then dorsal stream information. As most studies examine expertise differences and visual search behaviour in anticipation skills (Connor et al., 2018), or use a simulation of the environment (Hüttermann et al., 2019), there is the need to examine expertise differences in visual search behaviour in the execution of discrete skills in dynamic in-situ environments. However, currently there are very few studies have utilised these “live” game scenarios to understand visual search behaviour in discrete skill tasks (i.e., throwing a pass) in a semi-predictable sport environment (for exceptions, see Martell and Vickers, 2004; van Maarseveen et al., 2018). In rugby, an attacking player with the ball is constrained with only being able to pass the ball backward (as opposed to most other team sports where the ball can be passed in any direction) to a teammate. Due to this requirement, it is likely that the attacking player’s movement patterns and visual search behaviour will be constrained by the movement patterns and positioning of teammates when passing the ball, as opposed to other team sports (e.g., soccer and basketball). To fully understand how expertise influences visual search behaviour in this dynamic relationship between players, the current study collected visual search data using a mobile eye tracker in “live” attacking rugby scenarios. Within this setting, both experienced and novice rugby players were placed in attacking scenarios and were required to score a try against a defender. In this 2 vs. 1 game play, we created four scenarios of varying complexity, where players had to decide to take “the defender on” or “pass the ball.”

Based on emerging evidence that the relationship between expertise and visual search behaviour in-situ is different from simulated environments (Mann et al., 2021) we hypothesised that experienced rugby players would (1) demonstrate superior performance compared to NP (2) direct visual search behaviour for more time, and more often, toward areas related to the successful executing of the action (i.e., throwing a pass) compared to NP.

## Materials and Methods

### Participants

Twenty male participants were recruited to the study and split into two groups based upon their prior rugby union playing experience. The EP consisted of 10 participants (mean age 21.37 ± 1.76 years with 12.89 ± 1.86 years of experience) currently competing at a regional level and had previously represented their respective counties. All participants in the EP played in attacking positions (“backs” playing position 10–15) and were experienced with the attacking scenarios presented. The NP consisted of 10 university students (mean age 20.25 ± 1.91 years) who reported little to no previous rugby experience. The study was approved by Anglia Ruskin University Ethics Committee and the tenants of the Declaration of Helsinki were observed. Written informed consent was obtained from each participant prior to participation.

### Protocol

**FIGURE 1:**
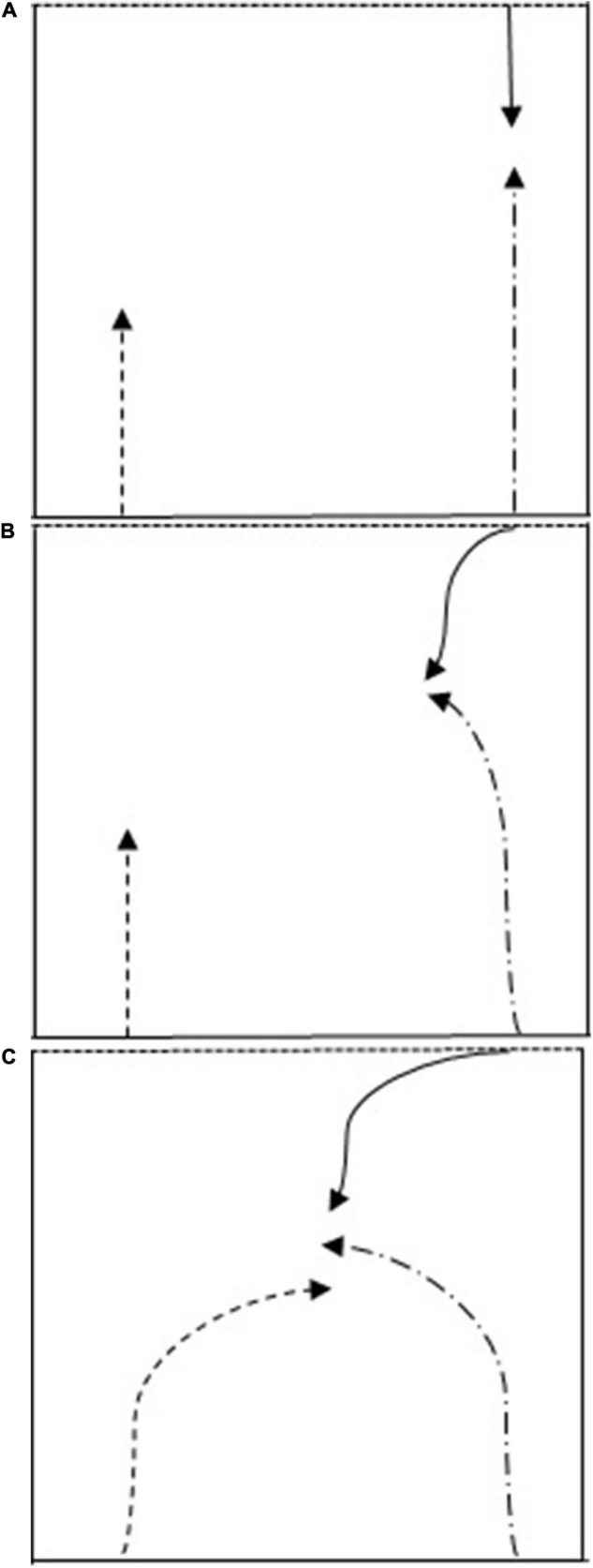
**(A)** Spin pass. The participant (Long Dash Dot arrow) runs toward the defender (Solid arrow). The ball is passed to the support player (Dash arrow) who runs toward and places the ball on the ground after the try line (Square dot line). **(B)** Take-Player-On. The participant (Long Dash Dot arrow) runs toward the defender (Solid arrow). Utilising speed and/or direction change, the participant attempts to run past the defender (passing on either side) and places the ball on the ground after the try line (Square dot line). **(C)** Switch and Dummy Switch. The participant (Long Dash Dot arrow) runs toward the defender (Solid arrow). The participant arcs their running angle to run laterally across the defender. The support player (Dash arrow) arcs their run around the back of the participant. Switch: The participant passes the ball to the support player, who runs toward and places the ball on the ground after the try line (Square dot line). Dummy Switch instead of the participant passing the ball to the support player, the participant feigns the pass and (through utilising speed and/or direction change) places the ball on the ground after the try line (Square dot line).

Participants completed a 2 (attacker) vs. 1 (defender) scenario in an area 10 m long and 8 m wide in an outdoor environment on natural turf. Participants (attacking player starting the trial with the rugby ball in their hands) were required to complete a self-paced task where the aim was to score a try. Four scenarios were developed (see Figure 1) involving passing the ball to their supporting attacking player, take the defender on or work with support player (Switch and Dummy switch). The trial was complete, either when a try was scored, the defender “tackled” the player with the ball or support player (defender was required to place both hands around the attacking players’ waist who was currently holding the ball cf. rules of Touch Rugby; World Rugby, 2009), or if the rugby ball was dropped or not passed adhering to the rules of rugby union (World Rugby, 2009).

Holding a size 5 rugby ball (Gilbert Barbarian match ball), participants initially stood with their back to the try line. Upon the verbal command “GO” (from the research assistant), the participant was required to turn around (counter-clockwise) and attempt to score a try using one of 4 different scenarios (Figures 1A–C). The participant always started with the ball in hand.

To avoid the support player becoming overly fatigued throughout data collection, one of three support players were used (same support player used for a participant’s entire data collection); regional level players, 13.46 ± 0.89 years playing experience. The same defender (15 years’ experience, playing at regional level) was used throughout the entire study. The defender was unaware of the scenario chosen by the participant and was given the instruction prior to each trial to “tackle the player with the ball and prevent the scoring of a try.” The defender and attackers always started from the same position.

Each participant was required to complete all 4 scenarios 3 times, in any particular order. For any novice participant who was unsure of a particular scenario, instruction and coaching points were delivered from an accredited level two Rugby Union Coach prior to data collection. All participants were given the opportunity to complete practice trials to become familiar with wearing the eye tracker. The defender was not present in the familiarisation period.

### Equipment

Eye movements of the participant were recorded using an SMI iViewETG head mounted mobile eye tracker (SensoMotoric Instruments Inc., Warthestr; Germany, Ver. 1.0) at 30 Hz with a spatial resolution of 0.1° and gaze position accuracy of 0.5°. The eye cameras had a gaze tracking range of 80° horizontally and 60° vertically where the high definition (HD) scene camera (1280 × 960 pixel, 24 Hz) had a tracking range of 60° horizontally and 46° vertically. Data from the eye tracker were recorded on a mini laptop (Lenovo X220, ThinkPad, United States) with iView ETG (Ver. 2.0) recording software installed. The laptop was placed in a backpack worn by the participant during testing. None of the participants reported that wearing the backpack affected their balance whilst completing the trial, however, some experienced participants reported that the laptop reduced their acceleration, impacting their ability to run away from the defender in Dummy Switch and Take-Player-On scenarios. A three-point calibration was performed to verify point of gaze before the trials. This calibration was verified after every trial to allow for post data collection recalibration.

### Data Analysis

Point of gaze data was analysed offline frame-by-frame with Begaze analysis software (SMI, Teltow, Germany Ver. 3.4). Each point of gaze in the real-time dynamic visual scene was mapped manually (frame by frame) to Areas of Interest (AOIs). Fixations were determined as four or more consecutive frames (120 ms) to an area of interest; a threshold consistent with previous research used to define a fixation (e.g., Williams et al., 1994). Each trial was tracked from the first frame where the participant began to turn their head after hearing the “GO” command. The end of the trial was identified in two different ways. For the Spin Pass and Switch scenarios, each trial was tracked up until the first frame whereby the ball had left the participant’s hands, being passed to the support player. With no pass occurring in the Take-Player-On and Dummy Switch scenarios, the end of the trial was instead identified when the participant had either run past the defender (i.e., beaten the defender), or when the player was tackled.

**FIGURE 2:**
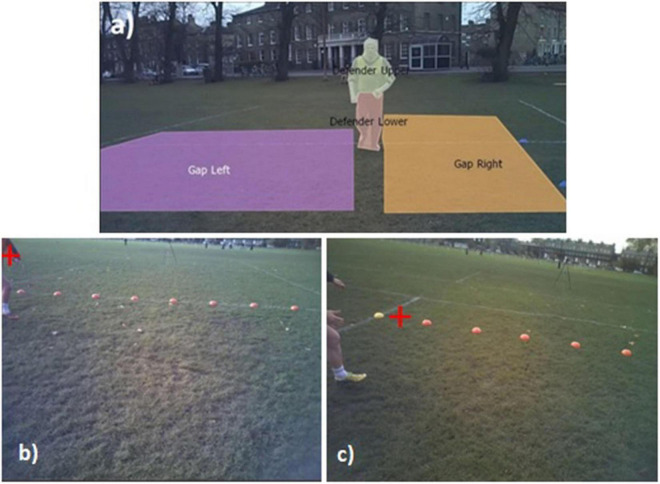
**(a)** Coding window used to track AOIs Gap Left, Gap Right, Defender (upper and lower). Illustration of fixation on **(b)** Support Player **(c)** Fixation Ahead Support Player.

The AOIs used defined key locations within the visual scene in relation to the action completed and comprised of Start of Trial, Direction of Travel, Turn Support-and-Defender, Defender,^1^ Support Player, Ahead Support Player, Gap Left and Gap Right (Figure 2).

*Start of Trial:* the beginning of the trial up until the point where the participant first fixated on the defender^2^.

*Direction of Travel;* when fixation was “visually anchored” immediately ahead of the individual and being carried along by the whole body movement; similarly termed travel gaze fixation (c.f. Patla and Vickers, 1997).

*Turn Support-and-Defender;* the period whereby the participant turned from looking at the defender to look at the support player or vice-versa.

The following variables were used to analyse eye tracking data;

1. Trial length (sec)– see description above.
2. Scan Rate (nr/sec)– the number of fixations per second.
3. Relative number of fixations on each AOI (%).
4. Relative fixation time on each AOI (%).

### Statistical Analysis

For the analysis of visual search data, from the 20 participants collected, data from 1 NP was below the acceptable tracking ratio of ≥ 90% (Vansteenkiste et al., 2014) and was not retained for statistical analysis; performance data (pass success and try outcome) was retained.

To assess inter-rater reliability of tracking visual search behaviour, a random selection of trials (10%) from four participants was coded by two researchers (MT and JL). An acceptable average intraclass correlation coefficient was reached when determining relative fixation length at Gap Right (*r* = 0.99) and relative fixation length at Defender (*r* = 0.99).

An initial analysis on the three trial repeats across all variables demonstrated no significant main effect of repetition, or repetition-by-group, or repetition-by-scenario interaction effect (*p* > 0.05). Data were subsequently averaged across trial repeats.

Separate 2 (experience level, groups) × 4 (scenario) mixed ANOVAs were run on all dependent variables related to visual search. Homogeneity of variance was checked using Levene’s test. All data were checked for Sphericity using Mauchly’s test. Level of significance was accepted at *p* < 0.05. *Post hoc* analyses, where appropriate, were performed using a Bonferroni correction. Effect sizes were calculated using Partial Eta squared $\left(\eta_{\rho }^{2}\right).$ Only main effects for experience level and experience*scenario interactions are reported to test our hypothesis related to expertise differences. Main effects of scenario are not reported and available from the corresponding author upon request.

A Chi-square test was used to analyse the total number of successful passes (Switch and Spin Pass) and the total number of successful tries (Switch, Spin Pass, Dummy Switch, and Take-Player-On) both between and within groups.

## Results

### Performance

Significantly more (χ^2^ = 18.47, *p* < 0.001) successful tries were completed in the EP (29 successful) compared to NP (14 successful). The increased number of successful tries was reflected in a significant difference between groups in number of successful passes (χ^2^ = 11.88, *p* = 0.001). The EP completed 29 successful Spin Passes, compared to 18 completed by the NP. There was no significant difference between groups in the number of successful Switch passes (*p* > 0.05, EP 20 successful Switch passes, NP 19 successful Switch passes).

### Trial Length and Scan Rate

There was no significant main effect of group on trial length [*F*(1, 17) = 1.99, *p* = 0.18, $\eta_{\rho }^{2}$ =0.105]. There was a significant group*scenario interaction effect [*F*(3, 51) = 2.83, *p* = 0.048, $\eta_{\rho }^{2}$= 0.143] where the EP were significantly quicker than NP at initiating the Spin Pass (EP 2.012 ± 0.125 s, NP 2.279 ± 0.203 s, *p* = 0.003, $\eta_{\rho }^{2}$= 0.418). Scan rate was not significantly different between groups [*F*(1, 17) = 0.37, *p* = 0.550, $\eta_{\rho }^{2}$= 0.021].

However, a significant group*scenario interaction for scan rate [*F*(3, 51) = 3.74 *p* = 0.017, $\eta_{\rho }^{2}$ =0.180] revealed that EP had a significantly higher scan rate compared to NP in the Take-Player-On scenario (EP 3.33 ± 0.57 nr/sec, NP 2.73 ± 0.57 nr/sec, *p* = 0.036, $\eta_{\rho }^{2}$ =0.234).

### Relative Time of Fixating on an Area of Interest

#### Fixation Time at the Defender

**FIGURE 3:**
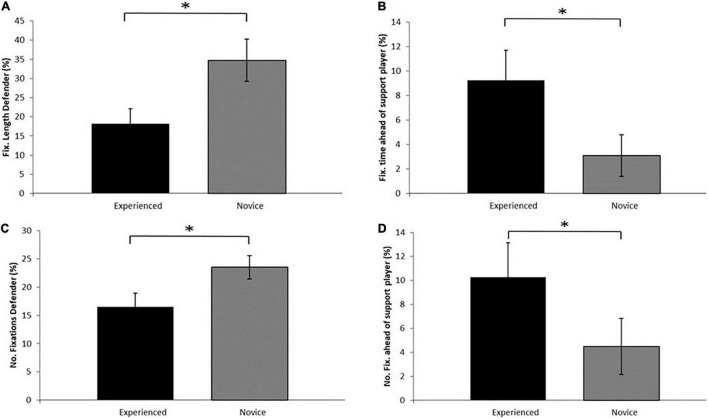
Relative fixation length **(A)** Defender **(B)** Ahead Support Player and Relative fixation number **(C)** Defender **(D)** Ahead Support Player for Experienced and Novice players. *p < 0.05.

There was a significant main effect of group *F*(1,17) = 10.38, *p* = 0.005, $\eta_{\rho }^{2}$ = 0.38 on the relative length of fixation on the defender where the NP spent significantly longer fixating on the defender compared to the EP (see Figure 3A). A significant group*scenario interaction effect *F*(3, 51) = 4.35 *p* = 0.008, $\eta_{\rho }^{2}$ = 0.204) identified that the NP spent significantly longer fixating on the defender compared to the EP in Spin Pass (NP: 48.78 ± 9.46%, EP: 25.66 ± 18.14%, *p* = 0.003, $\eta_{\rho }^{2}$ =0.408) and Take-Player-On scenarios (NP: 44.49 ± 11.78%, EP: 20.61 ± 10.87%, *p* < 0.001, $\eta_{\rho }^{2}$ =0.554, see Table 1).

**TABLE 1:** Visual search as a function of group (Experienced-EP, Novice-NP) and scenario (Spin Pass, Take-Player-On, Switch, and Dummy Switch).

	Experienced (EP)				Novice (NP)			
	Spin pass	Take player on	Switch	Dummy switch	Spin pass	Take player on	Switch	Dummy switch
Length of trial (s)	2.01(0.13)	2.15(0.18)	2.30(0.18)	2.31(0.17)	2.28(0.20)	2.26(0.25)	2.41(0.31)	2.29(0.31)
**Fixation time (%)**								
Trial start	30(9)	27(7)	26(6)	26(10)	27(5)	24(3)	24(5)	24(4)
Direction of travel	5(6)	15(9)	17(14)	22(13)	3(5)	9(7)	16(14)	16(10)
Defender	26(18)	20(10)	15(9)	12(12)	49(9)	44(12)	25(16)	21(18)
Support player	2(5)	2(2)	7(10)	6(8)	<1 (1)	<1 (1)	4(5)	4(3)
Ahead support player	15(12)	5(5)	10(10)	7(5)	3(3)	1(1)	4(6)	4(5)
Turn Def. and Sup.	11(5)	13(7)	14(8)	15(6)	9(5)	4(4)	17(6)	16(6)
Gap right	2(3)	5(4)	3(4)	2(2)	2(3)	8(6)	1(2)	<1 (1)
Gap left	9(6)	10(11)	6(5)	8(8)	8(8)	9(4)	9(8)	14(9)
**No. Fixations (%)**								
Trial start	19(5)	15(3)	17(4)	16(4)	18(2)	18(3)	15(4)	16(5)
Direction of travel	6(7)	15(10)	15(10)	17(7)	2(3)	9(6)	13(11)	14(8)
Defender	19(9)	20(8)	15(6)	12(8)	31(5)	31(6)	17(7)	15(8)
Support player	4(7)	2(3)	5(7)	6(6)	<1 (1)	<1 (1)	5(7)	6(5)
Ahead support player	17(9)	5(5)	10(6)	9(6)	6(5)	1(2)	6(7)	5(5)
Turn Def. and Sup.	14(6)	15(7)	21(5)	21(8)	18(6)	11(7)	20(8)	23(9)
Gap right	2(2)	8(6)	4(5)	2(3)	4(4)	13(8)	1(2)	<1 (1)
Gap left	12(7)	11(6)	12(8)	12(8)	10(6)	10(4)	11(8)	17(8)

*Data presented are the group mean (standard deviation).*

#### Fixation Time at, and Ahead of, the Support Player

There was no significant effect of group [*F*(1, 17) = 1.97, *p* = 0.179, $\eta_{\rho }^{2}$ = 0.104] or group*scenario interaction [*F*(2.22, 37.80) = 0.218, *p* = 0.827, $\eta_{\rho }^{2}$ = 0.013] on relative fixation time directly toward the support player.

However, there was a significant main effect of group *F*(1, 17) = 9.13, *p* = 0.008, $\eta_{\rho }^{2}$ = 0.349 on relative fixation time looking *ahead* of the support player. The EP fixated ahead of the support player longer compared to the NP (Figure 3B). There was no significant group*scenario interaction *F*(3, 51) = 2.11, *p* = 0.111, $\eta_{\rho }^{2}$= 0.110 on looking ahead of the support player.

#### Fixation Time at Gaps

There was no significant main effect of group [*F*(1, 17) = 0.038, *p* = 0.847, $\eta_{\rho }^{2}$ = 0.002] or significant group*scenario interaction effect *F*(1.60, 27.27) = 3.36 *p* = 0.059, $\eta_{\rho }^{2}$ =0.165 on relative fixation time to gap right. There was no significant main effect of group *F*(1, 17) = 0.35, *p* = 0.560, $\eta_{\rho }^{2}$= 0.020 or group*scenario interaction *F*(3, 51) = 1.44 *p* = 0.242, $\eta_{\rho }^{2}$= 0.078 on relative fixation time to gap left.

#### Fixation Time at the Direction of Travel

The relative length of fixation on Direction of Travel was not significantly affected by group *F*(1, 17) = 1.77, *p* = 0.201, $\eta_{\rho }^{2}$ =0.094, There was no significant group*scenario interaction *F*(3, 51) = 0.42 *p* = 0.738, $\eta_{\rho }^{2}=$ 0.024.

#### Fixation Time at Turn-Support-and-Defender

There was no significant main effect of group *F*(1, 17) = 0.96, *p* = 0.342, $\eta_{\rho }^{2}$ =0.053 on the relative length fixating Turn-Support-and-Defender. A significant group*scenario interaction effect *F*(3, 51) = 5.54 *p* = 0.002, $\eta_{\rho }^{2}$= 0.246 revealed that the Experienced group fixated Turn-Support-and-Defender longer (13 ± 7%) in Take-Player-On compared to Novice group (4 ± 4%, *p* = 0.002, $\eta_{\rho }^{2}$ =0.426).

### Relative Number of Fixations on an Area of Interest

#### Number of Fixations on the Defender

There was a significant main effect of *F*(1, 17) = 5.46, *p* = 0.032, $\eta_{\rho }^{2}$ = 0.24 on the relative number of fixations on the defender. The NP made significantly more fixations to the defender compared to NP (see Figure 3C). A significant group*scenario interaction *F*(3, 51) = 6.36 *p* = 0.001, $\eta_{\rho }^{2}$ = 0.272 identified that NP made more fixations to the defender compared to EP in the Spin Pass (*p* = 0.002, $\eta_{\rho }^{2}$ =0.434) and Take-Player-On scenarios (*p* = 0.005, $\eta_{\rho }^{2}$= 0.377, Table 1).

#### Number of Fixations on the Support Player

The relative number of fixations on the support player was not significantly affected by group [*F*(1, 17) = 0.66, *p* = 0.426, $\eta_{\rho }^{2}$ =0.038] and there was no significant group*scenario interaction [*F*(3, 51) = 0.83, *p* = 0.484, $\eta_{\rho }^{2}$ = 0.047].

There was a significant main effect of group *F*(1, 17) = 10.72, *p* = 0.004, $\eta_{\rho }^{2}$ =0.387 on the relative number of fixations *ahead* of the support player. The EP made significantly more fixations immediately ahead of the support player compared to NP (Figure 3D). There was no significant group*scenario interaction *F*(3, 51) = 2.04, *p* = 0.120, $\eta_{\rho }^{2}$ =0.107 for the relative number of fixations ahead of the support player.

#### Number of Fixations at Gaps

There was no significant main effect for group on the relative number of fixations on gap right *F*(1, 17) = 0.050, *p* = 0.825, $\eta_{\rho }^{2}$ =0.003. However, a significant group*scenario interaction effect [*F*(1.53, 25.94) = 4.77 *p* = 0.025, $\eta_{\rho }^{2}$ =0.219] highlighted that the EP fixated more frequently on gap right in Switch (*p* = 0.041, $\eta_{\rho }^{2}$ =0.201) and Dummy Switch conditions compared to NP (*p* = 0.044, $\eta_{\rho }^{2}$ =0.196, Table 1). No significant main effect of group *F*(1, 17) = 0.010, *p* = 0.921, $\eta_{\rho }^{2}$ =0.001 or group*scenario interaction effects *F*(3, 51) = 1.64 *p* = 0.192, $\eta_{\rho }^{2}$ =0.088 were identified for gap left.

#### Number of Fixations at Direction of Travel

The relative number of fixations on Direction of Travel was not significantly affected by group *F*(1, 17) = 1.76, *p* = 0.202, $\eta_{\rho }^{2}$ =0.094, There was no significant group*scenario interaction *F*(3, 51) = 0.19 *p* = 0.902, $\eta_{\rho }^{2}=$ 0.011.

#### Number of Fixations at Turn-Support-and-Defender

Relative number of fixation in Turn-Support-and-Defender was not significantly affected by group [*F*(1, 17) = 0.044, *p* = 0.836, $\eta_{\rho }^{2}$ =0.003] and there was no significant group*scenario interaction effect *F*(3, 51) = 0.845 *p* = 0.476, $\eta_{\rho }^{2}$ =0.047.

## Discussion

The current study investigated the adaptations which occur as a function of expertise in rugby player’s visual search behaviour when tasked with completing attacking scenarios. Differences in visual search behaviour between experienced and NP were observed in how long and how often players fixated immediately ahead of the support player in preparation of completing a pass. Concurrently, the mechanism of acquiring visual information in preparation and completion of the action (e.g., passing or taking on the defender) were significantly different. In line with our hypothesis, it was found that EP visually attended more often and for longer to areas related to opportunities for action (e.g., gaps on the side of the defender) where novices visually attended significantly longer to the defender, an area that could reflect a more allocentric perspective in motor response planning. These findings were accompanied by a superior performance of the EP compared to the NP.

By using “live” game scenarios we were able to create a representative design where rugby players completed an attacking phase of play. This allowed for the examination of expertise differences in visual search behaviour in both the planning and execution of the motor action. Previous studies (van der Kamp et al., 2008; Dicks et al., 2010; Mann et al., 2021) highlighted that the inclusion of the motor action influences the balance in deriving information from the dorsal and ventral visual system and therefore adopted visual search behaviour. As expertise differences reflect superior motor performance it is expected that the adopted visual search behaviour is also different. Indeed, our results indicate that superior performance of the EP was also evident in their visual search behaviour. This contradicts findings from Connor et al. (2018) where visual search behaviour of elite and novice rugby players was examined in anticipating evasion manoeuvres of attacking rugby players. Connor et al. (2018) did not identify significant difference in various visual search behaviour variables (i.e., viewing time at different AOIs) that could support the significant improvement in anticipation skills in the elite players. However, Connor et al. (2018) did not include a motor component in the anticipation of the evasion manoeuvres and therefore predominantly examined ventral stream related visual search behaviour, excluding dorsal stream influences. Particularly the ability to derive visual information through the dorsal stream underpins superior performance of experts.

Within scenarios which required the participants to pass the ball (Spin Pass and Switch scenarios), a number of simultaneous processes occur which inform processes made regarding stimulus-related variables (e.g., travelling velocity of the person to pass to) and response-related variables (e.g., distance required to pass the object, Lim, 2015), all of which are informed based upon an egocentric perspective where the acquired visual information reflects the position and motion of the body with respect to the environment (Davids et al., 2004). The distance the ball was required to travel (particularly in the Spin Pass scenario) and the speed the support player was travelling meant that passing the ball at the support player would result in the ball arriving behind them and the ball not being caught; alternatively, the support player would be required to slow down/stop running to catch the ball, increasing the likelihood of the defender running across to make a successful tackle. Instead, successful interceptive throwing/passing tasks result from fixating at the interception point (Lim, 2015), which in the current study was immediately ahead of the support player. Indeed, despite the importance of the participant acquiring positional information of the support player (i.e., ensuring the support player is not positioned in advance of your position) the length and time EP spent fixating the support player only accounted for a small proportion of the trial (Table 1). Instead, EP fixated longer and more frequently immediately ahead of the support player and acquired information of the support player from their periphery (e.g., Piras et al., 2014a,b) in comparison to the NP. By not directly fixating the support player (turning the head left to centralise the fovea on the support player), this also maximised the area in the visual scene which could still be perceived from the opposite side of the periphery, which contained other key information sources (e.g., location of the oncoming defender). In the current study, the NP did not adopt this strategy. The NP spent an equally small proportion of the trial fixating on the support player and immediately ahead of the support player (Table 1). This finding supports that the EP initiated the pass earlier in the phase of play in comparison to the novices. A subsidiary analysis of all the trials completed in the Spin Pass scenario, when collapsed across group, demonstrated the importance of fixating the interception point and not the support player when passing. A successful pass and try resulted from fixating significantly longer at the area ahead of the support player [pass *t*(41) = 2.65, p = 0.011, try *t*(45) = 2.630, *p* = 0.012], but this was not apparent when looking directly at the support player (*p* > 0.05).

Expertise specific differences in visual search behaviour where also apparent in examining the environment in guiding motor actions. The EP exploited gaps to the right of the defender significantly more than NP in the more complex Switch and Dummy Switch scenarios. Similarly, there were clear significant differences in the length and frequency of attending to the defender. NP visually attended significantly more time and more often to the defender in general and specifically in Spin Pass and Take-Player-On scenarios. Both these findings reflect how expertise influences the extraction of visual information with constant environmental constraints.

In both Switch and Dummy Switch scenarios, the support player was required to arc their run (from the left of the participant) behind the participant, eventually running into gap right (Figure 1C). To maximise the opportunity to score a try in these scenarios, the participant was required to create space for the support player to run into. Insufficient space between defender and attack/support player allowed the defender to “tackle” the ball carrier. During Dummy Switch and Switch scenarios only, EP fixated more frequently in gap right compared to NP. With no associated increase in time spent looking at gap right, it appears that EP were “checking” that sufficient space was being created for the support player to run into, something that NP did not do.

Concurrently to differences in exploiting the space around the defender it is equally important to acquire visual information regarding the movement and intentions of the defender. The defender was consistently and significantly more frequently (NP 23.5 ± 6.5 vs. EP 16.5 ± 7.7%) and for longer (NP 34.8 ± 17.4 vs. EP 18.2 ± 12.2%) visually attended to by the NP than the EP (see Figures 3A,C). The importance of directing visual search behaviour at the defender is supported by previous research which has reported that during 1-on-1 situations in rugby, experienced defending players focus on the pelvic region of the opponent (Brault et al., 2010), as this provides the most accurate cues relating to the opponent’s future running direction (Brault et al., 2012). This contrasts our findings and highlight the importance of task constraints on visual search behaviour (c.f. Rienhoff et al., 2016 for an overview of the influence of constraints on visual search behaviour). In our attacking scenarios, in contrast to defending scenarios in Brault et al. (2010)), visually attending to the defender was less important to our EP than our NP, specifically in Take-Player-On and Spin Pass scenarios. An explanation of this can come from the influence of expertise on dorsal and ventral visual systems. The increase in visually attending to the defender suggests that the NP use the defender to plan and guide their motor action based on defender movement and anticipation of defender movement (i.e., visual anticipation *via* the ventral stream). In contrast, the NP attend less to the defender but direct attention more to areas that guide their motor actions (e.g., area ahead of support player) reflecting a greater reliance on the dorsal stream. These marked differences in visual search behaviour as a function of expertise in a “live” scenario provides further support for the use of representative designs as these differences disappear in an anticipation video-based paradigm (Connor et al., 2018).

### Limitations

Within our study we measured visual search behaviour of rugby players, and novices, with a mobile eye tracker in live game scenarios. By comparing novices to EP our effects might be more pronounced then when inEP would have been used. Our intention was to provide evidence to where experts look to execute these tasks to support the adopted visual search behaviour of experts and how this could be positioned into a two-stream hypothesis. As such, a comparison to novices could provide this. However, the inclusion of a third group (i.e., inEP) could have enriched our findings. A concurrent challenge of using novice participants are differences in running speed and timings of passes, both influencing scenario completion time. These effects can exaggerate between group differences in visual search behaviour. We addressed this limitation by expressing visual search behaviour relative to trial time and therefore ensuring comparability between our novice and experienced participants.

## Conclusion

When completing attacking scenarios in rugby, differences in visual search behaviour are apparent as a function of expertise. Differences in visual search behaviour between experienced and NP related to the more refined awareness and functional understanding of the strategic role of space immediately ahead of the support player by experts. This space is exploited to guide motor action from an egocentric perspective in contrast to the novices who seem to rely more on information from defender movement and actions to build an allocentric perspective to predict their future movements.

Future research may wish to consider how to expedite player learning through cueing visual attention to key strategic areas during attacking play in rugby. Whilst approaches to and effectiveness off perceptual-cognitive skills training are varied (see Klostermann et al., 2015) and initial understanding of the adopted gaze behaviour of experts can form the foundations of a successful intervention. Such approaches are recommended to be designed with a representative design reflected in the inclusion of relevant motor actions and an information rich environment with a large variety of perceptual stimuli.

## Data Availability Statement

The raw data supporting the conclusions of this article will be made available by the authors, without undue reservation.

## Ethics Statement

The studies involving human participants were reviewed and approved by the Faculty of Science and Engineering Ethics Committee at Anglia Ruskin University. The patients/participants provided their written informed consent to participate in this study.

## Author Contributions

KP, IB, and MT contributed to conception and design of the study and wrote sections. MT and JL performed the data collection. KP, JL, PR, and MT contributed to data and statistical analysis and interpretation. MT wrote the first draft of the manuscript. All authors contributed to manuscript revision, read, and approved the submitted version.

## Conflict of Interest

The authors declare that the research was conducted in the absence of any commercial or financial relationships that could be construed as a potential conflict of interest.

## Publisher’s Note

All claims expressed in this article are solely those of the authors and do not necessarily represent those of their affiliated organizations, or those of the publisher, the editors and the reviewers. Any product that may be evaluated in this article, or claim that may be made by its manufacturer, is not guaranteed or endorsed by the publisher.

## Footnotes

1 In the initial tracking we separate the defender in Upper and Lower part (see Figure 2). In the reported results we combined these AOIs due to similarity in results.

2 Start of trial was mapped to derive trial length (i.e., start of action to end of action). Statistical analysis of Start of Trial are not reported.
